# Digitization path to improve ESG performance: A study on organizational perspectives

**DOI:** 10.1371/journal.pone.0313686

**Published:** 2024-12-04

**Authors:** Feifei Zhao, Zhipeng Han, Liguo Wang

**Affiliations:** 1 School of Economics and Management, Shandong Youth University of Political Science, Jinan, China; 2 Airport College, Shandong University of Aeronautics, Binzhou, Shandong, China; 3 School of Investment Engineering Management, Dongbei University of Finance and Economics, Dalian, China; Universidad Internacional de La Rioja, SPAIN

## Abstract

Digital technology development provides new opportunities for environmental, social, and governance (ESG) performance research to better evaluate firm ESG performance, improve decision-making efficiency, and enhance firm competitiveness. Therefore, under the background of digital economy, studying digitization mechanisms on ESG performance is of great theoretical and practical significance, which can help firms achieve better sustainable development and create more value for stakeholders. We use 3,827 listed A-share companies in China from 2003 to 2021 as the sample for our empirical research. Results show that digitization significantly improves ESG performance, and this conclusion remains valid after a series of robustness tests. Through mechanism analysis, we find that digitization improves ESG performance through organizational resilience and further reveal that organizational redundancy has a positive moderating effect between organizational resilience and ESG performance. According to our heterogeneity analysis, the marginal effects are stronger among listed firms with high market competition, in the East-Central region, in non-heavily polluting industries, and with standard audit opinions, without significant heterogeneity across the nature of equity. Our research provides a theoretical basis for digitization to drive ESG performance and ideas on how to improve the ESG performance of Chinese companies in the digital era.

## 1. Introduction

Environmental, social, and governance is a form of non-financial reporting by companies, in addition to financial reporting, to demonstrate their efforts and achievements in sustainability, social responsibility and good governance. In 2004, UNGC released a report entitled “Who Cares Wins”, in which the concept of ESG (Environmental, Social and Governance) was first introduced. Since then, ESG has gradually become a new value guideline in the business world, guiding companies to focus on environmental, social and governance performance while pursuing economic benefits. ESG is now an important criterion for the international community to measure the level of firm green sustainability [1]. Since China proposed the “Double carbon” (i.e., emission peak and carbon neutrality) strategy, ESG investment and related policies are attracting attention from all societal sectors, and the China Securities Regulatory Commission (CSRC) is planning ESG disclosure rules for listed companies in China to strengthen the fulfillment of firm ESG responsibility. ESG is not only a key criterion for assessing the overall company level but also is increasingly becoming an important tool for implementing China’s “Double carbon” strategy.

As the new round of technological revolution centered on big data, Internet of Things (IoT), and artificial intelligence computing is shifting from the introduction period to the expansion period, the digital economy and digitization process in China have significantly accelerated. Most scholars suggest that digitization has a positive impact on firm performance [2, 3]. In the long run, digitization also has social and environmental sustainability functions due to its contribution to energy sustainability, harmful gas emission reduction, and social welfare improvement [4]. As García-Muiña et al. (2021) [5] pointed out, the digitization of production processes not only enables the assessment of environmental impacts, but also has a crucial role in understanding the social performance of manufacturing companies.

Firms are gradually recognizing the importance of digitization and undertaking digital transformation, and the ESG concept is increasingly gaining consensus among institutional investors and the public. However, there are few studies on the relationship between firm digitization and ESG performance [6, 7]. Currently, the literature on ESG performance mainly focused on the economic consequences, such as financing ability, market performance, and investor preference [8]. Based on the above analysis, this paper takes firm digitization as an entrance point and provides an in-depth review of the literature related to firm digitization. It discusses the influence of firm digitization on ESG performance. Wang and Esperança (2023) [9] found that digital resources, digital organization, digital adoption, digital management, and firm competitiveness indirectly and positively affect ESG through firm market performance. Wu and Li (2023) [10] found that digital transformation will indirectly improve ESG performance by promoting corporate green innovation activities. He and Chen (2024) [11] suggested that firm digitization contributes to improved ESG performance by increasing the range of high-quality workforces and upgrading the skill levels of the existing workforce. Fang et al.(2023) [12] suggested that reducing agency costs within firms and improving the value of firms’ goodwill mediates the relationship between digitization and ESG performance. Cai et al.(2023) [13] found that firm digital transformation can alleviate financing constraints, increase analysts’ attention, and improve ESG performance. Jin and Wu (2024) [14] explored the effect between digital transformation and ESG performance from the perspectives of dynamic capability(absorptive capacity, adaptive capacity, and innovation capacity) and institutional environment. Liu et al. (2024) [8] found that digital transformation promotes ESG performance from three aspects: improving innovation performance, reducing information asymmetry, and optimizing corporate governance efficiency.

Although these studies have made some theoretical contributions from different perspectives,

it is found that the existing literature has not yet formulated a unified perception of the relationship between digitization and ESG performance. It is still important and meaningful to explore in depth the mechanisms of firm digitization and ESG performance in the context of the China.

In view of this, this paper argues that the existing literature has largely ignored the role of organizational resilience in the relationship between digitization and ESG performance. In addition, the current literature on organizational resilience focuses more on its contribution to innovation and sustainability, while few previous studies have directly demonstrated the mediating role of organizational resilience on digitization and ESG performance. Organizational resilience is the ability of an organization to withstand shocks, maintain core functions, and bounce back to recover from them [15]. It is not only the ability to bounce back and recover from adversity, but also the ability to grow and develop new business in the face of adversity [16]. Williams et al. (2017) [17] further argued that organizational resilience is developed from a set of “capabilities for durability”. Capabilities for durability are endowments of capabilities that an organization possesses that exist before an adversity event occurs and that drive the organization to adjust positively in response to the adversity event, specifically in the five dimensions of financial, behavioral, cognitive, emotion-regulation, and relational endowments [17]. The ultimate goal and achievement of building organizational resilience is to achieve long-term growth, high performance [18] and high rates of long-term growth based on long-term survival [19]—even if short-term gains may be compromised [20].

We select a sample of 3,827 listed A-share companies in China from 2003 to 2021 for our empirical study. This paper proves that the digitization significantly enhances ESG performance through a series of robustness and endogeneity tests. Our mechanism analysis reveals that digitization improves ESG performance through organizational resilience and further reveal that organizational redundancy has a positive moderating effect between organizational resilience and ESG performance.

The expected contributions of this study compared to previous studies are as follows. (1) This study examines the factors influencing ESG performance in the digital era, expanding the analysis of ESG-related antecedent variables. Although research on ESG performance is beginning to abound, most of the literature still focuses primarily on the role of ESG on firm development, while relatively little attention has been paid to the factors that influence it [10]. (2) This study innovatively explores the mediating path of digitization on ESG performance from the perspective of organizational resilience, which makes up for the lack of exploration of the current mechanism of digitization on ESG performance. It opens the “black box” of the impact of digitization on the ESG performance. As pointed out by Yu and Zhu (2022) [21], existing research lacks relevant research on ensuring the sustainable performance of resilient organizations through day-to-day management practices. We introduce organizational resilience into the study of the relationship between digitization and ESG performance to extend the applicability of organizational resilience. Meanwhile, this study tests the moderating role of organizational redundancy between organizational resilience and ESG performance, which extends the study of boundary conditions for the relationship between digitization and ESG performance. (3) This study provides additional evidence on the influence of firm digital scenarios and more accurately assesses the impact of digitization on ESG performance. We further explore the heterogeneity of the effect of digitization on ESG performance through subgroup regressions and find that the marginal effect is stronger among listed firms with high market competition, in the East-Central region, in non-heavily polluting industries, and with standard audit opinions, without any significant heterogeneity across the nature of shareholdings. The study of the economic and social effects of firm digitization is thus expanded and deepened.

## 2. Theoretical analysis and research hypothesis

### 2.1 Digitization and ESG performance

Based on new institutional theory, firm digitization attracts external attention and generates external institutional pressures, firms face legitimacy pressures and must improve ESG performance to meet the legitimacy needs of the external environment [22]. According to signaling theory, the application of digital technology as a positive market signal can help reduce the degree of information asymmetry, alleviate financing constraints, and protect expenditures that satisfy ESG requirements, as well as reduce agency problems, inhibit management’s myopia, focus on long-term green development, and enhance the willingness of companies to improve ESG performance [23]. Stakeholder theory suggests that digitization enriches the social and governance dimensions of corporate performance by increasing transparency and information dissemination, and by meeting stakeholder demands for corporate social responsibility and information accessibility [24].

Firm digitization is the process of applying digital technology in firms, the core of which lies in information collection, data processing, and digital technology application to assist decision making [25]. Its strictly technical significance has gradually developed into a major transformation process within and between organizations and societies [26]. Firm digitization not only reduces production costs and improves productivity but also reduces environmental pollution, realizes green growth, and promotes high-quality firm development in China.

First, in terms of the environment, firm digitization level can help improve the quality of the environment and improve the environmental performance of firms themselves. Digital twins, infinite convergence and digitization self-iteration, can significantly improve energy and material use efficiency, reduce carbon emissions in the production process, and further drive production and carbon emission optimization in the whole industrial chain. In addition, digital technology application can optimize the process flow through the automation upgrade in the production process and improve the digital energy management levels of firms themselves. Existing studies have found that firm digital transformation significantly reduces pollutant emissions, reduces wastes in the production process, and achieves energy saving and emission reduction, thus improving their own environmental performance [27, 28].

Second, on the social side, firm digitization, as a systemic strategic change, has an impact on multiple stakeholders to a certain extent. Firm digitization provides a fundamental digital vehicle to support the value creation of empowered firm stakeholders. On the one hand, as a new tool for social governance, data can effectively improve the ability to analyze and make decisions on data analysis and scheduling within the enterprise, and provide a new means to effectively deal with complex social issues. Digitization enables firms to quickly capture social public issues through digital technology and strengthen their own strategic orientation toward social responsibility [29]. In addition, digitization has the advantages of zero marginal cost, scale effect, and optimal allocation of resources, which help to solve the problem of information asymmetry between different governance subjects and improve the level of corporate social responsibility. On the other hand, digitization raises external attention and scrutiny, and growing accountability requires companies to establish extensive and detailed environmental monitoring and impact management systems [30]. As a result, firms seek further social reputation, which in turn, motivates them to discipline or correct their behaviors to be in line with social norms.

Finally, on the corporate governance side, firm digitization is conducive to reducing information asymmetry and improving rational analysis and decision making, thus positively influencing the way and effectiveness of corporate governance [31]. In addition, digitization can enhance ESG performance by detaching companies from their inherent mindset and making them more flexible in formulating business strategies and management structures, creating a virtuous circle pattern of corporate governance. In summary, we posit the following:

**Hypothesis 1 (H1): Firm digitization has a positive impact on ESG performance.**

### 2.2 Mechanisms in organizational perspective

With the increasing research on digitization in the theoretical community, scholars have begun to emphasize their focus on the study of the impact of digitization on organizational capability. Digitization can form new dynamic capabilities [32]. Organizational resilience, as a specific organizational capability (i.e., the ability of firms to anticipate, avoid, and respond to internal and external environmental disruptions), emphasizes recovery and healthy growth in the face of adversity [20]. Digital empowerment theory argues that empowered subjects are empowered by digital technology to eliminate their powerlessness under crises, achieve rapid responses to crisis changes, and enable them to acquire appropriate life skills and survival capabilities in crisis situations [33]. Dai and Fang (2024) [34] found that firm digitization plays a significant positive role in innovation performance, ESG performance, working capital management performance, organizational resilience, and corporate market competitiveness. Dubey et al. (2021) [35] demonstrated through an empirical study that big data technologies can leverage their data mining and analytics capabilities to have significant positive effects on supply chain resilience. Zhang and Huang (2024) [36] argued that digital transformation can enhance supply chain resilience, namely by reducing supplier and customer concentration to improve a firm’s ESG performance.

On one hand, firm digitization improves organizational stability and adaptability. First, firm digitization provides resources for the organization’s options for action and can facilitate the realization of collaborative and coordinated behaviours in practice, thus supporting the organization in developing and executing continuity plans [37], maintaining its core organizational attributes, avoiding or mitigating the damage caused by shocks, and demonstrating strong resilience to shocks. Second, digital technology helps organizations build a strong information processing capability, and can effectively alleviate the problem of information asymmetry. This enables firm to sense changes in the internal and external environments in a timely manner, thus monitoring and predicting potential risks or shocks and strengthening the organization’s anticipation of crises [38]. At the same time, digital technologies provide tools for companies to learn from past decisions and results, enabling reflection and learning and furthering organizational optimization and restructuring [37].

One the other hand, organizational resilience may have a positive impact on ESG performance. First, a good resilient system helps firms protect, remediate, and govern the environment. Rai et al. (2021) [39] posited three dimensions of organizational resilience, crisis prediction, organizational robustness and recoverability, which can lead to social and economic sustainability. Taking measures to promote social sustainability, such as protecting resources and reducing pollution, makes accessing resources and reducing costs easy for resilient organizations [39]. From the perspective of stakeholder theory, when firms pay more attention to establishing close and stable interest relationships with stakeholders to achieve sustainable development of the firm, the ESG performance of the firm will also be improved. Second, organizational resilience has a positive impact on corporate social responsibility (CSR). Firms with great organizational resilience tend to focus on long-term strategic goals and are likely to invest their redundant resources in social causes to build their organizational reputation. Finally, firms with great organizational resilience generally have many social capital and network connections, which are also positive for them to learn and absorb knowledge from external networks to improve their corporate governance, resilience, and ESG in a unified framework for consideration. In summary, we propose the following:

**Hypothesis 2a (H2a): Organizational resilience has a mediating effect between firm digitization and ESG performance.**

Unabsorbed redundant resources possessed by firms can help them accelerate the technological renewals of their products in competitive markets, thereby facilitating optimal resource allocation and enhancing firm efficiency, thus giving them competitive advantages in markets [40, 41]. Organizational redundancy can improve environmental performance by stimulating innovation [42], allowing firms to participate in pollution prevention strategies [43]. Symeou et al. (2019) [44] showed that firms’ failure to absorb redundant resources has a positive impact on their environmental performance. Xu et al. (2015) [45] showed that unabsorbed redundant resources positively affect social performance. Organizational redundancy, as a resource buffer for firms, helps them cope with unexpected crises and has a positive impact on corporate governance level.

Combined with the previous reasoning on the mediating role of organizational resilience, we argue that the higher the organizational redundancy, the greater the positive impact of organizational resilience on ESG performance. The reason is that when many redundant resources exist, organizations have room for resource adjustment and are likely to use excess resources for optimal resource allocation. Given that certain organizational resilience levels are necessary to achieve organizational requirements and deliver improved ESG performance, sufficient resources are needed to support firm decisions. On the contrary, when less organizational redundancy exists, even though organizations have certain resilience levels, it may be insufficient due to the lack of resource support for rebound and resilience, resulting in poor ESG performance. In summary, we propose the following:

**Hypothesis 2b (H2b): Organizational redundancy has a moderating effect between organizational resilience and ESG performance.**

## 3. Study design

### 3.1 Baseline model

We use time and individual two-way fixed effects (TWFE) model for estimation. In panel data, individuals not only have between-group (individual-to-individual) but also within-group (among each individual) disparities. Time fixed effects can address the problem of omitted variables that do not vary with individuals but vary over time. Individual fixed effects is used to capture differences among individuals who do not vary over time. TWFE considers time fixed effects and individual fixed effects to obtain accurate relationships. The specific formula is set up as shown in Eq (1).

$${ESG}_{i,t}=\alpha_{0}+\alpha_{1}Digit_{i,t}+\overset{n}{\underset{a=1}{\sum }}\beta_{a}controls_{i,t}+\mu_{i}+\lambda_{t}+\varepsilon_{i,t},$$ (1)

where _*i*_ represents each listed firm; _*t*_ refers to the year; *α*_*0*_ is a constant term; *ESG* is ESG performance; and *Digit* is the digitization degree; *controls* refer to each control variable; *ε*_*i*,*t*_ represents the random error term; *μ*_*i*_ is an individual individual fixed effects; and *λ*_*t*_ represents time fixed effects. The coefficient *α*_*1*_ of *Digit* is mainly observed in the model regression results to estimate the effect of digitization on ESG performance.

### 3.2 Data processing

Given the availability and timeliness of data, we select the data of listed A-share firms from 2003 to 2021 as the research sample. Data search strategy for this manuscript from the database in the S1 File. We process the data as follows: (1) excluding samples with missing variables; (2) excluding listed companies marked as st, st*, and pt; (3) tailoring all variables at the 1% level to reduce extreme value effects. The final data of 39,978 valid samples are obtained after processing.

### 3.3 Variable definition and description

#### 3.3.1 Dependent variable

At present, no unified international ESG rating system exists. Nevertheless, more than 600 rating agencies around the world have proposed their own representative definitions, among which the influential ones are Bloomberg, MSCI, Thomson Reuters, Refinitiv, and Sino-Securities Index. Some differences are observed in the characteristics, rating methods, and even products and services of different ESG rating agencies, but they are almost similar. Given that Sino-Securities Index started to rate the ESG performance of listed Chinese firms earlier to meet the data availability of this study, we adopt the ESG rating of Sino-Securities Index, referring to the study of Lin et al. (2021) [46]. The rating scale is divided into nine levels (AAA, AA, A, BBB, BB, B, CCC, CC, and C), and we assign a value of “9–1” to each of them; the higher the value, the better the ESG performance.

#### 3.3.2 Independent variable

Digitization refers to firm digital transformation by applying traditional management, operation, and service models to achieve the intelligence, automation, and science of management, operation, and service through digital technology application.

As a major strategy for high-quality development in the new era, digitization is likely to be reflected in a firm annual report, which is a summary and guidance. The usage of words in an annual report can reflect the strategic characteristic and future outlook of a firm, and to a large extent, the business philosophy promoted by the firm and the development path guided by this philosophy. Therefore, characterizing the degree of transformation of listed companies from the perspective of the frequency statistics of words related to “digitization” in their annual reports is feasible and scientific. Referring to Wu et al. (2021) [47] and Fang et al. (2023) [12], we use the text analysis function of Python to construct independent variable *Digit* in the following three steps: (1) summarize the specific keywords related to digitization based on the articles in the academic field and documents in the industrial field; (2) conduct the word frequency statistics of the annual reports of each listed firm in each year based on the keywords that have been mentioned, and after processing, obtain the panel data; (3) add 1 and take the natural logarithm to obtain the overall index of firm digitization, considering that this kind of word frequency data has typical “right bias” characteristics.

#### 3.3.3 Control variables

To make the regression results as close to reality as possible, we control for a number of other factors that may affect ESG performance, including: firm size (*Size*), measured by taking the natural logarithm of the number of employees of a firm plus one; listing age (*ListAge*), measured by taking the natural logarithm of the current year minus the listing year plus 1; revenue growth rate (*Growth*), management fee rate (*Mfee*), leverage (*Lev*), stockholding concentration (*Top10*), measured by the shareholding ratio of the top 10 shareholders; and book-to-market ratio (*BM*).

The descriptive statistics of the above variables are presented in Table 1.

**Table 1:** Descriptive statistics.

Type	Variable	*N*	Minimum	Maximum	Mean	Standard deviation
Dependent variable	ESG	39,978	0.000	9.000	4.564	3.112
Independent variable	Digit	39,978	0.000	6.176	0.758	1.203
Control variable	Size	39,978	1.946	13.220	7.619	1.293
	FirmAge	39,978	0.000	4.159	2.773	0.416
	Growth	39,978	−1.309	134607	4.116	677.4
	Mfee	39,978	−0.757	382.100	0.108	2.089
	Lev	39,978	0.007	9.699	0.430	0.213
	Top10	39,978	0.013	1.012	0.591	0.152
	BM	39,978	0.003	30.560	1.060	1.297

## 4. Regression results and discussion

### 4.1 Regression results of the baseline model

Based on the baseline model constructed above, this section examines the quantitative impact of digitization on ESG performance, and the regression results are shown in Table 2. Columns (2)–(4) are the regression results with control variables gradually added on top of the regression results in Column (1). Column (4) shows the regression result of the baseline model. The coefficient of independent variable *Digit* in all regression results is significantly positive at the 1% level, indicating that digitization has a significant improvement effect on ESG performance. With the gradual addition of control variables, the coefficient and standard error of the core independent variable *Digit* tend to stabilize. Fit degree R^2^ also stabilizes after optimization, which not only indicates that no omitted variable problem exists in the baseline model but also supports the rationality that the model contains TWFE.

**Table 2:** Regression results of the baseline model.

Variable	(1)	(2)	(3)	(4)
	ESG	ESG	ESG	ESG
Digit	0.029***	0.019***	0.019***	0.019***
	(0.007)	(0.007)	(0.007)	(0.007)
Size		0.149***	0.151***	0.145***
		(0.008)	(0.008)	(0.008)
FirmAge		0.056	0.067	0.090
		(0.055)	(0.055)	(0.057)
Growth		−0.000***	−0.000***	−0.000***
		(0.000)	(0.000)	(0.000)
Mfee			−0.006	−0.006
			(0.005)	(0.005)
Lev			−0.060	−0.087*
			(0.043)	(0.047)
Top10				0.056
				(0.056)
BM				0.022***
				(0.006)
_cons	4.542***	3.262***	3.239***	3.176***
	(0.007)	(0.157)	(0.158)	(0.169)
Observations	39,961	39,961	39,961	39,961
R^2^	0.920	0.921	0.921	0.921
Individual-FE	Yes	Yes	Yes	Yes
Period-FE	Yes	Yes	Yes	Yes

Note: Robust standard errors at the firm level are in parentheses

***, **, and * indicate significance at the 1%, 5%, and 10% levels, respectively.

So far, we cannot infer that H1 holds and must further verify the robustness of the regression results of the baseline model.

### 4.2 Robustness analysis

#### 4.2.1 Instrumental variable analysis

In general, certain factors cause the endogeneity problem and thus perturb the regression results. Finding a suitable instrumental variable for independent variable *Digit* can effectively solve the endogeneity problem. We choose the number of smartphone users (*Phone*) as the instrumental variable due to the fact that, on the one hand, the release of mobile smart apps by firms is an important part of digitization. The number of smartphone users also affects the conversion efficiency of this link, which satisfies the relevance principle of the instrumental variable. On the other hand, the number of smartphone users has no direct logical relationship with firm ESG performance, which satisfies the exogeneity principle of the instrumental variable.

We perform two-stage least squares (2SLS) regression using instrumental variable *Phone*, and the regression results are presented in Table 3, with Columns (1) and (2) showing the first- and second-stage 2SLS regression results, respectively. The coefficient of instrumental variable *Phone* is significantly positive in the first-stage regression, and the coefficient of *Digit* is significantly positive in the second-stage regression. In addition, the Cragg–Donald Wald F statistic is 168.090, exceeding the 10% threshold of the Stock–Yogo test (16.380), thus, no weak instrumental variable problem exists. The *p*-value of the Kleibergen–Paap rk LM statistic is 0.000, which is less than 1%, indicating that the original hypothesis of “under-identified instrumental variables” is rejected at the 1% level. The above regression results and tests suggest that the baseline regression results are still robust after excluding the endogeneity problem.

**Table 3:** Robustness analysis results.

Variable	(1)	(2)	(3)	(4)	(5)
	2SLS (first-stage)	2SLS (second-stage)	DID	Heckman model	System GMM
	Digit	ESG	ESG	ESG	ESG
Digit		0.820***	0.033**	0.076***	0.120***
		(0.121)	(0.015)	(0.010)	(0.045)
Phone	0.001***				
	(0.000)				
L.ESG					0.501***
					(0.033)
Observations	31940	31940	39961	39978	35642
R^2^	0.698	0.868	0.921		
Control	Yes	Yes	Yes	Yes	Yes
Individual-FE	Yes	Yes	Yes	Yes	Yes
Period-FE	Yes	Yes	Yes	Yes	Yes

Note: Robust standard errors at the firm level are in parentheses

***, **, and * indicate significance at the 1%, 5%, and 10% levels, respectively.

#### 4.2.2 Difference-in-difference (DID) model

We choose DID model to further verify the robustness of the baseline regression. First, we construct individual grouping dummy variables *treat* and period grouping dummy variables *post*, assign *treat* to 1 for the sample with a digitization keyword in the annual report and assign it to the experiment group; otherwise, assign it to 0 and to the control group; second, we assign *post* to 1 for the year in which the digitization keyword appears in the annual report of the experiment group and the subsequent years; otherwise, we assign it to 0. Subsequently, the cross product term of *treat* and *post* is defined as the new independent variable *Digit_DID* and put into the baseline model for regression.

We also did joint significance test for leads and lags, this test is consistent with the original hypothesis of F test in multiple regression, i.e. all variables are equal to 0. The F-statistic for the joint significance test for leads is 0.768 and its p-value is 0.615, indicating that all periods ex ante are not significantly different from 0, while the F-statistic for the joint significance test for lags is 5.324 and its p-value is 0.000, indicating that all periods ex post are significantly different from 0. This is further evidence of passing the parallel trend test.

Columns (3) Table 3 show the regression results of the DID model. Digitization still significantly improves ESG performance, suggesting that the regression results of the baseline model are robust.

#### 4.2.3 Heckman two-stage model

Social science data are for the most part non-randomly assigned, making the role of selection models in research increasingly important. However, the importance of the exclusionary constraint variables is often overlooked, and arbitrary or no exclusionary constraint variables can make the models often weak and lead to very unrobust conclusions. In addition, sample selection bias may also occur. Sample selection problems may be due to problems in the sample collection process or self-selection of the study population, leading to endogeneity problems.

The unbiased estimation of multiple regression depends on the correct setting of the functional form, otherwise, the functional form misspecification (FFM) may lead to biased estimates. The propensity score matching (PSM) model reduces the dependence on the functional form by matching and relaxes the linearity assumption of the multiple regression model, thus alleviating the FFM problem. However, the PSM approach does not fundamentally address the endogeneity problem caused by selection bias or omitted variables, and cannot replace methods such as Heckman and IV for solving the problems of self-selection and omitted variables; moreover, in cases where the "common support assumption" cannot be satisfied or is far-fetched, the PSM systematically excludes the FFM problem. In addition, if the "common support" assumption is not satisfied or is far-fetched, the PSM systematically excludes samples lacking controls, thus making the sample less representative and affecting the external validity of the results.

Therefore, we choose the Heckman two-stage model to address the above problem, using the Probit regression model and calculating the inverse mills ratio (IMR) based on the regression results in the first step and bringing the IMR into the model for regression in the second step [48]. As shown in column (4) of Table 3, the coefficients of the independent variables *Digit* in the regression results of Heckman two-stage model remain significantly positive. We can now infer that H1 is valid, i.e., digitization improves ESG performance.

#### 4.2.4 Generalized method of moments

ESG performance may be serial in time, and we choose the dynamic panel data (DPD) model to consider the lag term of ESG, as shown in Eq (2).

$$ESG_{i,t}=\alpha_{0}+\alpha_{1}Digit_{i,t}+\alpha_{2}ESG_{i,t-1}+\overset{n}{\underset{a=1}{\sum }}\beta_{a}controls_{i,t}+\mu_{i}+\lambda_{t}+\varepsilon_{i,j,t},$$ (2)

where *ESG*_*i*,*t-1*_ denotes one period lag of *ESG*_*i*,*t*_. In the DPD model, given that the lagged term of the dependent variable is used as the independent variable, which may lead to the correlation of the independent variable with the disturbance term, traditional estimation methods may fail for the above situation. Therefore, we refer to Blundell and Bond (1998) [49] for parameter estimation using generalized method of moments (GMM). There are two commonly used GMMs, different GMM and system GMM, Bond (2002) argues that GMM estimation results are valid when the coefficient of the lagged one-period term of the dependent variable in GMM estimation lies between the coefficient values estimated by ordinary least square (OLS) and fixed effects (FE). We did the test accordingly and found that only the coefficient of the first-order lagged term of the dependent variable ESG in the system GMM (0.501) lies between its OLS (0.654) and FE (0.254) estimation, so we concluded that the system GMM is valid here. We believe this may be due to the fact that the model contains explanatory variables that do not vary over time and the differential GMM is unable to estimate these variables efficiently, coupled with the weak instrumental variable problem of the different GMM, so the system GMM is considered more efficient [50].

The results are presented in Table 3. Columns (5) show the estimation results of system GMM, where the coefficient of independent variable *Digit* remains significantly positive. In addition, the *p*-values of AR(1) for the Arellano–Bond test of system GMM is 0.018, less than 1%. Meanwhile, the *p*-values of AR(2) is 0.672, more than 10%. Therefore, the random error term of the first-order difference equation is first-order autocorrelated, rather than second-order and later autocorrelated, proving that DPD model setting is appropriate. The *p*-values of the Sargan tests for system GMM is 0.921 which exceed 10%, suggesting that no over-identification problem exists. The above estimation results and test results indicate that the regression results are still robust after considering the dynamic effects.

## 5. Mechanism analysis

The results of the baseline model regressions and a series of robustness tests confirm that digitization can improve ESG performance, and we further test the mechanisms through which this improvement works. We use stepwise regression to explore the mechanism in depth [51, 52]. Drawing on Zhao et al.(2010) [53], we improve the model on the basis of the previous stepwise regression method model and set it as follows:

$$mediator_{i,t}=\gamma_{0}+\gamma_{1}Digit_{i,t}+\overset{n}{\underset{a=1}{\sum }}\delta_{a}controls_{i,t}+\mu_{i}+\lambda_{t}+\nu_{i,t},$$ (3)

$$ESG_{i,t}=\alpha_{0}+\alpha_{1}Digit_{i,t}+\alpha_{2}mediator_{i,t}+\overset{n}{\underset{a=1}{\sum }}\beta_{a}controls_{i,t}+\mu_{i}+\lambda_{t}+\xi_{i,t},$$ (4)

where *mediator* represents the mediating variable; other variables are consistent with the baseline model setting. We focus on whether independent variable *Digit* coefficient *γ*_*1*_ in Eq (3) and *mediator* coefficient *α*_*2*_ in Eq (4) are significantly positive; if so, then it indicates a positive mediation effect.

A second-stage moderated mediation is suggested in the hypothesis, which is a mediating process by which the mediating variable connects with the dependent variable and is influenced by the moderating variable [54]; therefore, we draw on Hayes (2017) [55] and the second-stage moderated mediation model, as presented in Eq (5).

$$\begin{array}{l} ESG_{i,t}=\alpha_{0}+\alpha_{1}Digit_{i,t}+\alpha_{2}mediator_{i,t}+\alpha_{3}moderater_{i,t}\\ \;+\alpha_{4}mediator_{i,t}\times moderater_{i,t}+\sum_{a=1}^{n}\beta_{a}controls_{i,t}+\mu_{i}+\lambda_{t}+\xi_{i,t} \end{array},$$ (5)

where *mediator* represents the mediating variable, *moderater* is the moderating variable, and other variables are consistent with the baseline model setting. Arguing that the mediator effect changes with the moderating variable is crucial [56], so for the moderating effect, we focus on whether the coefficient *α*_*4*_ of the cross-product term *mediator × moderater* in Eq (5) is significantly positive; if so, then it indicates that *moderater* has a positive moderating effect on the mediator effect.

Based on the previous theoretical analysis, we select organizational resilience as mediator variable, and organizational redundancy as moderator variable. Two variables are measured using the following methods.

(1) Organizational resilience (*Resil*). Organizational resilience is the ability of an organization to adapt to changes in the external environment and to continuously improve, resulting in high long-term performance growth and low financial volatility. To measure organizational resilience, Ortiz-de-Mandojana and Bansal (2016) [20] proposed a methodology that conceptualizes organizational resilience as a two-dimensional structure with high long-term performance growth and low financial volatility. First, the metric used to measure the long-term performance growth of a firm is the three-year average sales growth rate, as the average growth rate is a better indicator of longevity than the year-to-year growth rate. Second, financial volatility is measured as the stock return volatility, measured as the standard deviation of monthly individual stock returns over a one-year period, considering the reinvestment of cash dividends. Finally, long-term performance growth is used as a positive indicator, whereas financial volatility is used as a negative indicator to synthesize an organizational resilience indicator using the entropy value method to measure organizational resilience. We draw on this method to measure organizational resilience.

(2) Organizational redundancy (*Redu*). Organizational redundancy refers to the additional resources that organizations must achieve, which can help them cope with uncertain future circumstances. Financial redundancy, as available organizational redundancy, can usually be measured by determining the quick ratio [57–59]. The quick ratio is the ratio of a firm’s current assets to its current liabilities, which can be used to reflect the financial position of the firm and thus its financial redundancy level. Accordingly, we use the quick ratio as a proxy variable for organizational redundancy.

Table 4 reports the regression results based on the above four equations. The regression results in Columns (1) and (2) for Eqs (3) and (4) show that the coefficients of independent variable *Digit* are significant at the 1% level with coefficients of 0.003, indicating that digitization in turn significantly motivates organizational resilience. The estimated coefficients of organizational resilience (*Resil*) is significant at the 5% level with coefficients of 0.014, suggesting that organizational resilience improves ESG performance.

**Table 4:** Regression results of mechanism analysis.

Variable	(1)	(2)	(3)
	Resil	ESG	ESG
Digit	0.003***	0.014**	0.013**
	(0.000)	(0.007)	(0.007)
Resil		1.537***	1.003***
		(0.150)	(0.155)
Resil×Redu			0.236***
			(0.044)
Redu			−0.018***
			(0.004)
_cons	−0.267***	3.587***	3.618***
	(0.012)	(0.173)	(0.173)
Observations	39,961	39,961	39,961
R^2^	0.700	0.921	0.922
Control	Yes	Yes	Yes
Individual-FE	Yes	Yes	Yes
Period-FE	Yes	Yes	Yes

Note: Robust standard errors at the firm level are in parentheses

***, **, and * indicate significance at the 1%, 5%, and 10% levels, respectively.

Columns (3) show the regression results of Eq (5), where the coefficient of the cross product term *Resil×Redu* in Column (3) is significant at the 1% level with a coefficient of 0.236, indicating that the second-stage mediation of organizational redundancy (*Redu*) on organizational resilience (*Resil*) has a positive moderating effect.

The Sobel test *p*-values for all three mediating effects are less than 0.05, and the Bootstrap test results of 500 random samples show that the confidence interval of indirect effect does not contain 0, indicating that the mediating effect holds and is robust. H2a and H2b are thus confirmed.

## 6. Heterogeneity analysis

Although we argue that digitization significantly improves ESG performance, we must analyze the variations of effects across different types of listed firms to further refine the variability, diversity, and patterns of effects. We further explore the heterogeneous effect of digitization on ESG performance through group regressions to examine and discuss below aspects.

Our subgroup regressions are categorized into five groups: degree of market competition, location of office, whether belonging to a heavily polluting industry, nature of shareholding, and whether having a standard audit opinion. Among them, the degree of market competition is measured by the Herfindahl index, the larger its value, the higher the concentration of the industry, the lower the degree of competition. Whether belonging to a heavily polluting industry based on the Management List of Environmental Verification Industries for Listed Firms established by the Ministry of Environmental Protection in 2008 and the Guide to Environmental Information Disclosure for Listed Firms in 2010, combined with the Guidelines on Industry Classification for Listed Firms revised by CSRC in 2012. The regression results are shown in Table 5.

**Table 5:** Regression results for grouping.

	(1)	(2)	(3)	(4)	(5)	(6)	(7)	(8)	(9)	(10)
	Degree of market competition		Location of office		Whether belonging to a heavily polluting industry		Nature of shareholding		Whether having a standard audit opinion	
	High	Low	East central	Western	Yes	No	SOE	Non-SOE	Yes	No
	ESG	ESG	ESG	ESG	ESG	ESG	ESG	ESG	ESG	ESG
Digit	0.018**	0.025	0.020***	0.018	0.020	0.018**	0.042***	0.034***	0.017**	0.012
	(0.007)	(0.023)	(0.007)	(0.021)	(0.018)	(0.008)	(0.010)	(0.009)	(0.007)	(0.077)
_cons	3.316***	1.673***	3.245***	2.518***	3.017***	3.130***	2.220***	3.869***	3.322***	6.203**
	(0.179)	(0.559)	(0.185)	(0.456)	(0.320)	(0.211)	(0.196)	(0.287)	(0.172)	(2.664)
Observations	35845	4036	34486	5446	12121	27804	16335	23172	38904	763
R^2^	0.921	0.917	0.919	0.932	0.931	0.918	0.957	0.886	0.922	0.950
Control	Yes	Yes	Yes	Yes	Yes	Yes	Yes	Yes	Yes	Yes
Individual-FE	Yes	Yes	Yes	Yes	Yes	Yes	Yes	Yes	Yes	Yes
Period-FE	Yes	Yes	Yes	Yes	Yes	Yes	Yes	Yes	Yes	Yes

Note: Robust standard errors at the firm level are in parentheses

***, **, and * a at the 1%, 5%, and 10% levels, respective

Based on the above regression results, combined with the practice, we get the following conclusions:

(1) In highly competitive markets, enterprises must innovate and increase efficiency to maintain competitiveness. Digital transformation plays a key role in this process by optimizing resource allocation, enhancing operational efficiency, and improving customer interaction, directly impacting ESG performance. For instance, the use of big data and artificial intelligence technologies can help enterprises accurately forecast market demand and optimize inventory management, reducing overproduction and its associated resource wastage, while also enhancing customer satisfaction and reducing environmental impacts. Additionally, digital supply chain management systems can increase the transparency and traceability of the supply chain, enhancing corporate social responsibility.

(2) Enterprises in the Eastern and Central regions benefit from relatively mature digital infrastructure and government support, providing a favorable external environment for digital transformation. Regional policies tend to support technological innovation and digital upgrading, allowing enterprises to utilize these advantages by implementing smart manufacturing and Internet of Things technologies to improve production efficiency and product quality, reduce energy consumption, and decrease waste output. Furthermore, government policies on data openness also promote data sharing and cooperation among enterprises, strengthening collective efforts in social responsibility and environmental protection.

(3) Enterprises in non-heavy pollution industries, which are subject to fewer environmental regulations, can more easily focus on breakthroughs in social and governance aspects through digital means. These enterprises can enhance social responsibility by implementing digital human resource management systems that improve talent management and employee satisfaction. Moreover, digital transformation can enhance corporate governance transparency, such as through the implementation of electronic meeting records and decision-making processes, thereby increasing stakeholder trust and governance levels.

(4) National policies have a significant impact on the digital transformation and ESG performance of enterprises. Both SOEs and non-SOEs are guided by government policies on digital technology promotion and ESG standardization. This policy consistency enables both types of enterprises to receive similar resources and support in digital transformation. With the spread of digital technology, the difference in technological infrastructure between SOEs and non-SOEs is gradually shrinking. Both types of enterprises can improve their ESG performance through technological means such as cloud computing, big data, and artificial intelligence. Therefore, the driving role of digital transformation at the technological level shows similarity in both.

(5) For enterprises with audit opinions, digital transformation is not only a tool for enhancing efficiency but also essential for improving transparency and compliance. These enterprises implement comprehensive Enterprise Resource Planning (ERP) systems and compliance management software to ensure their operations meet environmental and social responsibility standards, while also enhancing the quality and accuracy of external reporting. Through this approach, enterprises not only meet regulatory requirements but also build stronger trust among investors and consumers.

## 7 Discussion

The empirical analysis conducted in this study underscores the proposition that digital transformation significantly enhances Environmental, Social, and Governance (ESG) performance. The robustness tests reaffirm the validity of these findings, thus establishing a reliable link between digital transformation and improved ESG outcomes. Through a mechanism analysis, it was determined that digital transformation facilitates ESG performance improvements by enhancing organizational resilience. Furthermore, it was revealed that organizational redundancy positively moderates the mediating effect of organizational resilience on ESG performance.

Digital transformation’s impact on enhancing organizational resilience can be attributed to several factors. Firstly, digital technologies enable better data collection, management, and analysis capabilities, which in turn support more informed decision-making and risk management. These capabilities are crucial for organizations to respond dynamically to environmental and social challenges, aligning with governance requirements more efficiently [34, 39]. Secondly, digital tools facilitate improved communication and collaboration among stakeholders, which is essential for fostering transparency and accountability within organizations [37].

The positive moderation by organizational redundancy suggests that having excess resources and capacities can further enhance the resilience provided by digital transformation. This redundancy allows organizations to experiment with new technologies and processes without jeopardizing their core operations, thus providing a buffer that supports innovative practices and enhances adaptability in rapidly changing environments [44, 45].

The heterogeneity analysis reveals that the positive impact of digital transformation on ESG performance is particularly pronounced in environments characterized by high market competition, non-heavy pollution industries, the Eastern and Central regions, and within non-state-owned enterprises with audit opinions. These findings can be interpreted as follows:

In highly competitive markets, organizations are compelled to innovate continually and improve operational efficiencies to sustain their market positions. Digital transformation offers the tools needed to achieve these goals while also enhancing ESG performance through more sustainable practices and better stakeholder engagement; These industries typically face less stringent environmental regulations, allowing them more flexibility to implement digital technologies that can improve social and governance aspects more so than environmental ones. This is particularly beneficial for boosting aspects of ESG that pertain to employee relations, corporate governance, and community engagement; Eastern and Central Regions: The availability of advanced technological infrastructure and more supportive governmental policies in these regions facilitate smoother digital transformations. This geographical advantage allows enterprises in these areas to integrate digital solutions more effectively, leading to more substantial improvements in ESG performance; The presence of audit opinions often implies a higher level of scrutiny regarding transparency and compliance. Digital transformation in these enterprises enhances data accuracy and availability, which improves compliance and transparency—key components of the governance dimension of ESG. Digitization transformation exhibits a significant ESG-enhancing effect in both SOEs and non-SOEs, indicating that there is no significant heterogeneity in the nature of equity.

This research confirms that digital transformation serves as a crucial lever for enhancing ESG performance across various organizational contexts. The insights gained emphasize the importance of adopting digital strategies tailored to specific industry and regional characteristics to maximize ESG benefits. Moreover, organizations should consider strategic investments in organizational redundancy to bolster resilience and take full advantage of digital transformation’s potential to improve ESG performance. Future research could explore the long-term impacts of digital transformation on ESG performance across different economic cycles and further investigate the roles of other moderating factors.

## 8 Conclusion

Although scholars have begun to explore the mechanism and effect of digitization on ESG performance, the “black box” has not yet been opened, and the boundary of influence has not yet been fully revealed. Therefore, this paper conducts an empirical study on the impact of digitization on ESG performance, clarifying its internal mechanism from the perspective of organizational resilience, and revealing the relationship between organizational resilience and ESG performance under the condition of organizational redundancy.

We use 3,827 listed A-share companies in China from 2003 to 2021 as the sample for our empirical research. This paper proves that the digitization significantly enhances ESG performance through a series of robustness and endogeneity tests. Our mechanism analysis reveals that digitization can enhance ESG performance through organizational resilience, and organizational redundancy has a positive moderating effect between organizational resilience and ESG performance. According to the conclusions of our heterogeneity analysis, the marginal effect of digitization on ESG performance enhancement is stronger among listed firms with high market competition, in the East-Central region, in non-heavily polluting industries, and with standard audit opinions, without any significant heterogeneity across the nature of shareholdings.

### 8.1 Management enlightenments

Based on the above conclusions, we propose three practical suggestions. First, firms should seize the opportunities brought about by the digital economy and accelerate the process of firm digital transformation by deeply integrating the fast-growing new generation of digital information technology with the traditional production, research and development, management and marketing of firms. Second, firms must strive to build resilient organizations and effectively orchestrate their own resilience networks with external resources. Firms continue to cultivate internal strength while actively seeking external complementary resources and not absorbing redundant resources, which can significantly improve their dynamic resilience and ESG performance. Third, government should strongly advocate and actively promote the ESG concept by incorporating environmental and energy-saving requirements into the early stages of product design and development, and guiding firms to develop a variety of programs, including smart systems to reduce environmental impacts, energy management technologies, energy conservation and emissions reduction. In addition, government should strengthen the construction of laws and regulations in areas related to corporate data and information disclosure, and improve the construction of credit supervision mechanisms to avoid the drawbacks of data falsification brought about by digitization.

### 8.2 Limitations

There are certain limitations in this study. First, our sample is drawn from listed A-share Chinese companies and does not cover firms from other countries, so the generalizability of our findings should be further investigated. Future researches are invited to study our findings in various contexts. We suggest a comparative study of firms in developing and developed countries may yield more insightful conclusions and insights. Second, we only explore one mechanism and boundary condition of the relationship between firm digitization and ESG performance. There is still room for further deepening the study on the mechanism of firm digitization affecting ESG performance. Third, the need for digitization is different for firms with different life cycles, and we argue that subsequent research could explore the impact of firm digitization on ESG performance from the perspective of firm life cycles.

## Supporting information

S1 File (PDF)
