# Supplementary material for: Digitization path to improve ESG performance: A study on organizational perspectives
Source: PLoS One. 2024 Dec 4;19(12):e0313686. doi: 10.1371/journal.pone.0313686 (PMC11616825; doi:10.1371/journal.pone.0313686)
Supplement: S1 File — (PDF) [file pone.0313686.s001.pdf]

Data search strategy for this manuscript from the database

Content

1. Most of the data for listed firms from China Stock Market & Accounting Research (CSMAR) Database 2

2. Listed firms' annual report raw data from CNINFO Database 4

3. ESG data from Wind Database 5

1. Most of the data for listed firms from China Stock Market & Accounting Research (CSMAR) Database

Step 1: Open the official CSMAR Database website (https://data.csmar.com/) and log in to your account.

Step 2: Go to Data Center - Corporate Research Series - Financial Statements

Step 3: The objectives of this operation are:

(1) 2003 ~ 2021, A-share, non-ST, consolidated statement, annual report

(2) Balance sheet (total assets, total liabilities, net investment property, net fixed assets, etc.)

(3) Income statement (total operating income, total operating expenses, etc.)

(4) Cash flow statement (cash paid to and for employees, cash flow from operating activities, etc.)

Step 4: First the time setting

Step 5: Then “Code Selection” - “All A Shares” - “Non-ST”

Step 6: Next, “Conditional Screening” - only retain “Consolidated Statements, only retain” Annual Report “The purpose of this step is to eliminate other sub-statements and duplicate statements. This step is very critical, equivalent to a pre-screening of the data has been carried out, you can save trouble for the subsequent data cleaning.

Step 7: Field settings, take the balance sheet as an example, select the "Total Assets, Total Liabilities " and other required fields.

Step 8: The last step, download the data to the mailbox, or directly download data to the local

Step 9: In the same way, get the fields for the income statement and cash flow statement and download them.

2. Listed firms' annual report raw data from CNINFO Database

Step 1: The URL is: http://www.cninfo.com.cn/new/index

Step 2: Click on the list of Search Box to bring up the options. Enter the code or pinyin initials or abbreviated Chinese characters and the options will pop up by themselves

Step 3: Check this box for Annual Report and tap Confirm. Clicking on the list, Search Box will bring up the window for selecting the date

Step 4: Date Selection. In this new pop-up box you can select the start date and end date according to your needs. Click on the leftmost text item to quickly select the date. Once set, click on the “Search” button and you will be redirected to the next web site

Step 5: Disclosure Page. Click on the annual report you want from the list of disclosure pages. Your browser will automatically open the PDF file of the clicked annual report.

Step 6: Click on the PDF reading page of the announcement to download can be downloaded.

3. ESG data from Wind Database

Step 1: First open the Wind database page, the URL is: https://www.wind.com.cn/, enter the main interface of the software, select and click on the main functional area “data” item;

Step 2: Then click on the “data” below the sub-projects “Export Data”;

Step 3: Finally, select the type of data you need to export to complete the operation.
